# Based on TransRes-Pix2Pix network to generate the OBL image during SMILE surgery

**DOI:** 10.3389/fcell.2025.1598475

**Published:** 2025-05-21

**Authors:** Zeyu Zhu, Peifen Lin, Lingling Zhong, Qing Wang, Jingjing Xu, Kang Yu, Zheliang Guo, Yicheng Xu, Taorong Qiu, Yifeng Yu

**Affiliations:** ^1^ Ophthalmic Center, The Second Affiliated Hospital, Jiangxi Medical College, Nanchang University, Nanchang, China; ^2^ Center of Ophthalmic, Heyou Hospital, Foshan, China; ^3^ School of Mathematics and Computer Sciences, Nanchang University, Nanchang, China; ^4^ Fuzhou Experimental School, Fuzhou, China; ^5^ Jiangxi Medical College, Nanchang University, Nanchang, China

**Keywords:** artificial intelligence, generative adversarial networks, opaque bubble layer, small-incision lenticule extraction, complication

## Abstract

**Aim:**

Generative adversarial networks (GANs) were employed to predict the morphology of OBL before femtosecond laser scanning during SMILE.

**Methods:**

A retrospective cross-sectional analysis was conducted on 4,442 eyes from 2,265 patients who underwent SMILE surgery at the Ophthalmic Center of the Second Affiliated Hospital of Nanchang University between June 2021 and August 2022. Surgical videos, preoperative panoramic corneal images, and intraoperative OBL images were collected. The dataset was randomly split into a training set of 3,998 images and a test set of 444 images for model development and evaluation, respectively. Structural similarity index (SSIM) and peak signal-to-noise ratio (PSNR) were used to quantitatively assess OBL image quality. The accuracy of intraoperative OBL image predictions was also compared across different models.

**Results:**

Seven GAN models were developed. Among them, the model incorporating a residual structure and Transformer module within the Pix2pix framework exhibited the best predictive performance. This model’s intraoperative OBL morphology prediction demonstrated high consistency with actual images (SSIM = 0.67, PSNR = 26.02). The prediction accuracy of Trans-Pix2Pix (SSIM = 0.66, PSNR = 25.76), Res-Pix2Pix (SSIM = 0.65, PSNR = 23.08), and Pix2Pix (SSIM = 0.64, PSNR = 22.97), Pix2PixHD (SSIM = 0.63, PSNR = 23.46), DCGAN (SSIM = 0.58, PSNR = 20.46) was slightly lower, while the CycleGAN model (SSIM = 0.51, PSNR = 18.30) showed the least favorable results.

**Conclusion:**

The GAN model developed for predicting intraoperative OBL morphology based on preoperative panoramic corneal images demonstrates effective predictive capabilities and offers valuable insights for ophthalmologists in surgical planning.

## Introduction

Myopia, the most prevalent refractive error, emerges as a significant public health concern due to its rising global incidence (Baird et al., 2020; Medina, 2022). Laser refractive surgery, a widely used treatment, has been proven to enhance visual quality safely and effectively, significantly improving quality of life and work performance in individuals with myopia (Kim et al., 2019; Wilson, 2020). Small Incision Lenticule Extraction (SMILE), a relatively novel femtosecond laser technique, offers several advantages over traditional Laser-Assisted *in Situ* Keratomileusis (LASIK). However, it is technically demanding and associated with a steep learning curve (Lin et al., 2024; Zhao J. et al., 2023). During the early learning phase, surgeons may encounter unforeseen complications, which can negatively affect surgical outcomes (Wan et al., 2021; Titiyal et al., 2017).

A common intraoperative complication in femtosecond laser photofracture of corneal tissue is the formation of opaque bubble layers (OBL), caused by the accumulation of air bubbles between corneal layers (Zhu et al., 2024). This condition may complicate lenticule separation during surgery or increase the complexity, potentially delaying postoperative vision recovery (Zhu et al., 2024; Yang S. et al., 2023). While previous studies have identified several risk factors for OBL formation (Yang S. et al., 2023; Son et al., 2017; Ma et al., 2018), no comprehensive model currently exists to predict the morphology of OBL. The development of such a predictive model would significantly improve surgical decision-making accuracy and enhance the safety of SMILE procedures.

The creation of the lenticule during laser scanning is a crucial step in SMILE surgery. This process, fully automated and controlled by machine, is critical (Teo and Ang, 2024). If complications arise during this phase, surgeons typically need to halt the surgery promptly and use alternative techniques or adjustments to resolve the issue (Wan et al., 2021). This not only poses a significant challenge to the surgeon’s skills but also increases patient anxiety, potentially affecting both the postoperative visual acuity and recovery (Yang S. et al., 2023; Titiyal et al., 2018). For minimizing these risks, pre-laser-scanning anticipation and identification of potential intraoperative complications are essential, especially in the suction-initiated step. Identifying any abnormalities during this phase enables surgeons to interrupt and restart the procedure without affecting the laser scanning. Research has shown that the major risk factors for OBL during SMILE surgery are associated with special corneal parameters (Yang S. et al., 2023; Son et al., 2017; Ma et al., 2018). An important question arises: Can artificial intelligence (AI) technology predict the morphology of OBL by analyzing the panoramic corneal images captured during the negative pressure suction phase of SMILE surgery?

With the rapid development of AI, it has increasingly become an impotant auxiliary tool in the filed of ophthalmology, demonstrating substantial potential in improving clinical management and workflows (Zheng et al., 2021; Xu and Yang, 2023; Yang W.-H. et al., 2023). In the field of image prediction, Generative adversarial networks (GANs), a key area within AI, offer substantial advantages in processing high-dimensional data and images, showing great promise in automated medical analysis, particularly for managing large, complex datasets related to human diseases (Saeed et al., 2021; Gong et al., 2021; Waisberg et al., 2025). Compared to other models, such as Conditional Diffusion Models or Autoregressive Models, GANs can directly model image-to-image mapping through adversarial training, which is suitable for tasks such as intraoperative OBL prediction that require detail preservation (Waisberg et al., 2025). Pix2Pix is a universal image-to-image translation model in GANs (Kim and Chin, 2023; Zhang et al., 2022; Abdelmotaal et al., 2021). However, when it was used in medical image generation, it often resulted in blurred and distorted outputs, and it was difficult to capture details, and the generated image lacks texture (Zhang et al., 2022; Kim et al., 2025). While the residual structure helps preserve shallow and high-frequency information across layers (Wang et al., 2017; Park et al., 2019), and the Transformer network enhances global understanding through its attention mechanism (Chen et al., 2021; Cao et al., 2021).

To address this, the study incorporated a combination of residual structure and Transformer module into the Pix2Pix to predict the morphology of OBL during SMILE surgery, providing crucial intraoperative support for surgeons. Given the increased difficulty when OBL forms at the posterior interface of the lenticule during laser scanning, this study focused primarily on analyzing OBL during this critical phase (Son et al., 2017; Titiyal et al., 2018). The model allows surgeons to implement timely interventions, such as promptly releasing negative pressure and adjusting surgical parameters. These measures can effectively reduce OBL incidence, thereby minimizing its negative impact on both the surgical process and postoperative visual recovery. This approach holds significant practical value for enhancing the quality and safety of SMILE surgery.

## Materials and methods

### Research object

This retrospective cross-sectional study was conducted in strict adherence to the Declaration of Helsinki and received approval from the Ethics Committee of the Second Affiliated Hospital of Nanchang University (Approval No. 2024086). It is registered with ClinicalTrials.gov (Identifier: NCT06577012). The study included patients who underwent SMILE surgery at the Ophthalmic Center of the Second Affiliated Hospital of Nanchang University between June 2021 and October 2022.

Inclusion criteria were as follows: (1) age between 18 and 45 years; (2) Corrected Distance Visual Acuity (CDVA) of 16/20 or better; (3) preoperative spherical equivalent (SE) ≥ −10.0 diopters; (4) relatively stable refractive error, with annual changes of less than 0.50 diopters over the past 2 years; (5) no contact lens use in the 2 weeks preceding surgery.

Exclusion criteria were as follows: (1) presence of ocular conditions other than myopia and astigmatism, such as keratoconus, severe dry eye, uncontrolled glaucoma, visually significant cataracts, or a history of ocular trauma; (2) prior ocular surgery; (3) history of systemic diseases that could compromise surgical outcomes or patient safety, including psychiatric disorders, severe hyperthyroidism, systemic connective tissue diseases, or autoimmune diseases.

### Surgical procedure

In this study, all patients underwent SMILE surgery for the correction of myopia and astigmatism. The procedures were performed by two experienced surgeons with 10 and 20 years of refractive surgery experience, respectively, each having performed thousands of procedures including PRK, FS-LASIK, and SMILE.

To minimize the risk of infection, patients were prescribed 0.3% gatifloxacin eye gel starting 3 days before surgery. Fifteen minutes prior to surgery, the nurse administered surface anesthesia using 0.5% proparacaine hydrochloride eye drops, with one drop every 5 min for a total of two drops. During the procedure, a femtosecond laser system (Carl Zeiss, VisuMax, Germany) was used. The laser settings were as follows: pulse energy of 135 nJ, spot and track spacing of 4.5 μm, corneal cap thickness between 100–120 μm, and a cap diameter of 7.5 mm. The femtosecond laser performed sequential cuts in the following order: initial posterior lenticule cut, followed by the lenticule side cut, anterior lenticule cut, and cap side cutting. The corneal micro-incision was made at the 2 mm mark at the 12 o’clock position, with an angle of 90°. The transition zone for astigmatism treatment was set to the default value of 0.1 mm (Table 1). During the procedure, the surgeon stabilized the eyeball using microscopic toothed forceps with the left hand, while employing a lenticule separation spatula with the right hand to sequentially separate the anterior and posterior interfaces of the lenticule. Once these steps were completed, the lenticule was extracted, followed by the removal of the eyelid speculum, marking the end of the surgery.

**TABLE 1:** SMILE surgical parameters.

Surgical parameters	Range
Wavelength/nm	1,053
Pulse duration/fs	400
Pulse emission frequency/kHz	500
Optical zone/mm	6.5
The transition zone for astigmatism treatment/mm	0.1
Cap diameter/mm	7.5
Cap thickness/μm	100-120
The line and spot separations/μm	4.5
Cap side cut angle/°	90
Incision width/mm	2
Energy/nJ	135

Postoperative care began on the first day after surgery, with the following regimen: 0.3% gatifloxacin eye gel four times daily for 1 week; 0.1% flumetholone eye drops four times daily for 4 weeks, with weekly frequency reductions; and sodium hyaluronate eye drops four times daily for 4 weeks.

### Data set building

#### Data acquisition

The panoramic corneal view during SMILE surgery and the laser scanning image of the posterior lenticule interface were captured from the SMILE surgical video in the VisuMax system.

The OBL area was measured according to the methods described in previous literature (Zhu et al., 2024; Yang S. et al., 2023; Son et al., 2017). Adobe Photoshop 2020 software (Adobe Systems, San Jose, CA) was used, and the OBL area was defined as the percentage of pixels that are two standard deviations (SD) brighter than the average background, with the corneal region selected by the elliptical marquee tool. To ensure measurement accuracy and consistency and reduce software operation errors, each measurement was independently completed by three senior surgeons and cross-checked.

#### Data preprocessing

**FIGURE 1:**
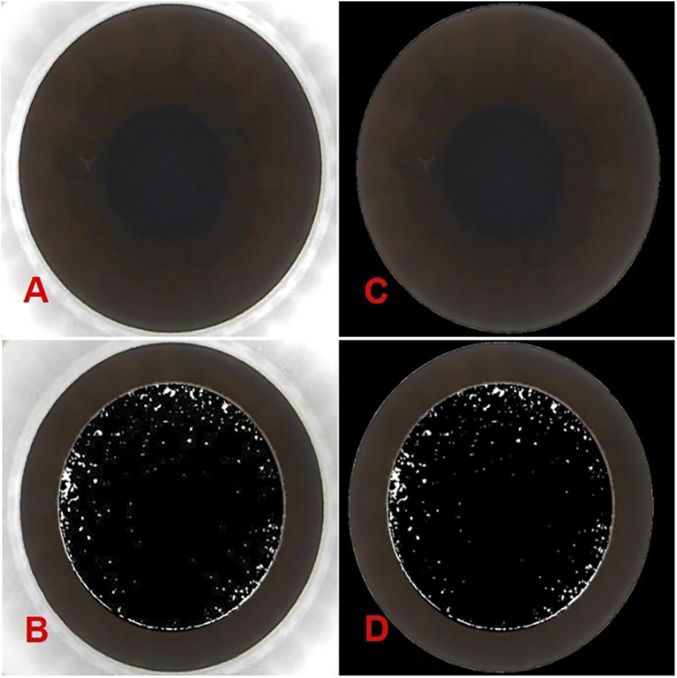
The panoramic corneal view processed using OpenCV within Python. **(A)** show the original panoramic views of the corneal captured from the VisuMax storage system (Carl Zeiss, Germany). **(B)** show the intraoperative OBL image processed by PS. While **(C,D)** display the processed panoramic views of the corneal and the intraoperative OBL image processed by PS after Python-based processing).

As the images obtained from the femtosecond laser system (Carl Zeiss, VisuMax, Germany) include both the cornea and portions of the surgical instrument, the focus for OBL prediction is solely on the corneal region. Consequently, it is essential to systematically remove non-corneal elements from the images to minimize potential errors. The images were initially processed using OpenCV within Python, employing binarization and closing operations to distinguish the corneal and non-corneal regions. Next, the Canny algorithm was applied for edge detection to identify and trace the image boundaries. A polygon approximation method was then used to smooth the contours, followed by ellipse fitting to determine the optimal fitting ellipse. The area within the ellipse was marked as the corneal region, and the regions outside this ellipse were masked in black to achieve segmentation. These preprocessing steps were applied consistently to both preoperative and postoperative images. The segmented images are presented in Figure 1.

The dataset was randomly divided into a training set (n = 3,998) and a test set (n = 444) using a random split method, ensuring that the network was trained and tested on different subsets of the data.

#### Model construction

The task involves generating an intraoperative image of the posterior interface, including the OBL area, from the panoramic corneal image captured before laser scanning. To tackle this image-to-image challenge, an image generation model that integrates a transformer block and a residual structure into the Pix2Pix framework was developed. This model leverages the Pix2Pix architecture to map the input image to the output image and enhances the network’s deep feature extraction capabilities by incorporating a residual structure. Additionally, the transformer block, with its self-attention mechanism, is adept at capturing long-range dependencies within the sequence, which helps maintain global consistency and structural integrity during the image generation process.

**FIGURE 2:**
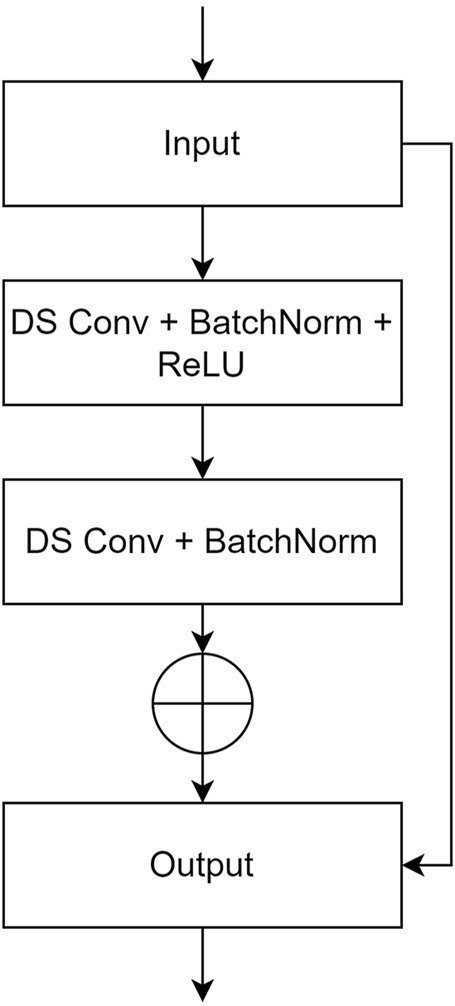
The user-defined residual structure diagram. (DS Conv: depthwise separable convolution; BatchNorm: batch normalization).

**FIGURE 3:**
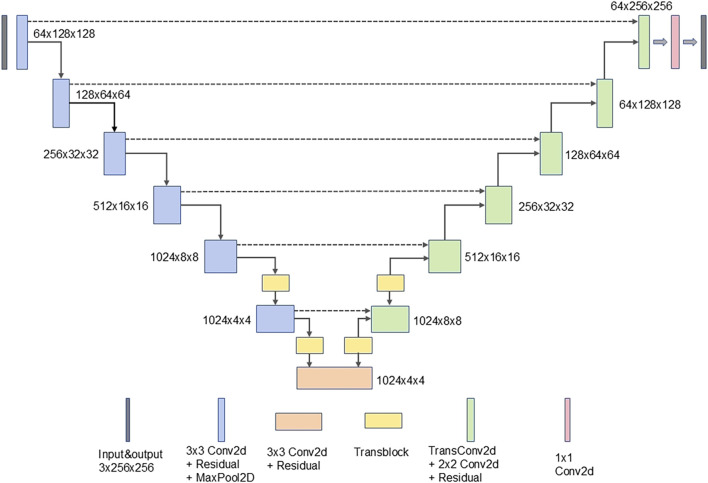
The complete generator network.

In this study, a deep UNet network was constructed as the generator, using Pix2Pix as the foundational framework. The model integrates a residual structure with depthwise separable convolution and incorporates several transformer blocks. To improve the model’s feature extraction abilities and enhance image generation performance, the UNet network was modified by integrating a custom-designed residual structure into the conventional convolutional blocks in both the encoder and decoder of the UNet-128 model. This modification increased the model’s depth by adding two layers and expanded the maximum channel capacity from 512 to 1,024. Moreover, to mitigate the impact of the increased number of layers on computational efficiency, depthwise separable convolution was incorporated within the residual structure, replacing traditional convolution. This adjustment reduced the model’s parameter count to one-third of the original while also lowering computational complexity. To further enhance the model’s ability to comprehend corneal images at a holistic level, transformer modules were introduced after the fifth and sixth layers of the generator’s encoder. Symmetrical transformer modules were added at corresponding positions within the decoder. The residual structure and the complete generator network are illustrated in Figures 2, 3.

**FIGURE 4:**
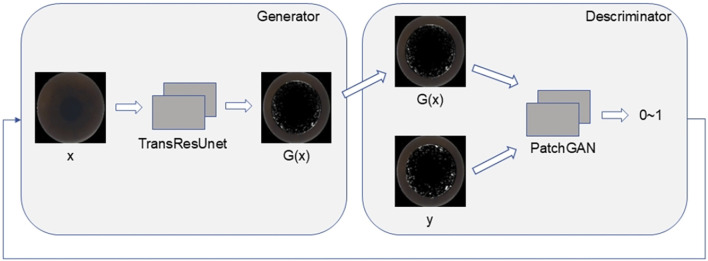
The TransRes-Pix2Pix integrated network structure.

The discriminator in this study utilizes the PatchGAN model, which operates by first concatenating the generated predicted image with the real image along the channel dimension. After passing through a convolution layer, the combined data undergoes further convolution processing. The PatchGAN output is then mapped to a single-channel two-dimensional matrix, maintaining the resolution of the original input. Each matrix element represents a similarity score for the corresponding patch, indicating the probability that the local area at that location is authentic or fabricated. Unlike PixelGAN, PatchGAN does not classify individual pixels but evaluates multiple local areas (patches), enabling it to capture rich texture and contextual information. This enhances the model’s ability to assess the similarity between the generated intraoperative image and the real image. The comprehensive network structure of both the generator and discriminator is illustrated in Figure 4.

Several optimization strategies were employed during model training. Binary Cross-Entropy Loss (BCELoss) served as the adversarial loss function, quantifying the discrepancy between the generated and real images within the discriminator. This mechanism improves the visual quality of the generated images, making them more similar to the real images (Ho et al., 2022). Additionally, L1Loss was applied to assess the pixel-level difference between the generated and real images, guiding the model to accurately match the content composition of the real images while enhancing clarity. The Adam optimizer was deployed for both the generator and discriminator to optimize the respective loss functions and expedite model convergence (Qu et al., 2023; Zadeh and Schmid, 2021).

#### Evaluation indicators

To thoroughly evaluate the performance of the generated images, multiple evaluation metrics were used. The Structural Similarity Index (SSIM) was employed to assess the structural similarity between the generated and real images. SSIM captures critical visual elements such as brightness, contrast, and structural integrity, ensuring that the generated image closely mirrors the real one. The SSIM index is calculated using the following formula:

$$SSIM\left(x,y\right)=\frac{\left(2\mu_{x}\mu_{y}+C_{1}\right)\left(2\sigma_{xy}+C_{2}\right)}{\left(\mu_{x}^{2}+\mu_{y}^{2}+C_{1}\right)\left(\sigma_{x}^{2}+\sigma_{y}^{2}+C_{2}\right)}$$ (1)

In formula 1, *x* and *y* are represent the generated image data and the real image data respectively. The values $\mu_{x}$ and $\mu_{y}$ are the mean values of x and y, while $\sigma_{x}$ and $\sigma_{y}$ denote their respective standard deviations. The covariance between x and y is represented by $\sigma_{\text{xy}}$ . The constants $C_{1}={\left(k_{1}\mathrm{MAX}\right)}^{2}$ and $C_{2}={\left(k_{2}\mathrm{MAX}\right)}^{2}$ are introduced to avoid division by zero, with $k_{1}$ and $k_{2}$ default values of 0.01 and 0.03, respectively. MAX refers to the maximum value of x and y.

The second metric, Peak Signal-to-Noise Ratio (PSNR), is widely employed to assess reconstruction quality in image processing. It quantifies the pixel-level difference between the predicted and real images. The PSNR is calculated using the following formula:

$$PSNR=20\cdot \log_{10}\left(\frac{\mathrm{MAX}}{\sqrt{MSE}}\right)$$ (2)

In formula 2, MAX represents the maximum value of the generated image data, while MSE refers to the mean squared error between the generated and actual image data.

#### Experimental environment

The experimental environment was set up using Python 3.8, PyTorch 2.2.2, and CUDA 12.2. The server configuration included an AMD R9 7950X processor, 64 GB of RAM, and an NVIDIA GeForce RTX 3090 Ti graphics card with 24 GB of video memory.

### Statistical methods

IBM SPSS Statistics 26.0 (IBM Corp., Armonk, NY, USA) was utilized for statistical processing and data analysis. All measurements are presented as mean ± standard deviation ( $\bar{x}\pm s$ ). Count data are presented as n (%).

SSIM evaluates the structural similarity between two images, with a value closer to one indicating greater similarity. In contrast, PSNR assesses image reconstruction quality by comparing the peak signal power to the noise power, with a higher PSNR indicating better reconstruction quality.

## Results

The study included 2,265 patients, corresponding to 4,442 eyes, all of which successfully underwent lenticule separation and removal during surgery. The average patient age was 21.88 ± 5.32 years. The mean preoperative spherical equivalent (SE) was −4.28 ± 1.83 D, and the average area affected by OBL was 3.26% ± 0.64%. Detailed surgical parameters are presented in Table 2.

**TABLE 2:** Preoperative general data of patients ( $\bar{x}\pm s$ , *n*%).

Parameters		Values
Age/y		21.88 ± 5.32
Gender/(n, M%)		3026 (68.12)
Eyes/(n, R%)		2259 (50.84)
CDVA/(logMAR)		0.00 ± 0.08
SE/D		−4.28 ± 1.83
Spherical/D		−3.96 ± 1.63
Cylinder/D		−0.63 ± 0.57
IOP/mmHg		15.81 ± 3.51
CCT/μm		545.97 ± 27.57
Corneal curvature	K1/D	42.36 ± 1.37
	K2/D	43.52 ± 1.45
Corneal diameter/mm		11.61 ± 0.44
Lenticule thickness/μm		91.42 ± 30.61
RST/μm		334.61 ± 37.66
Relative vertical position of posterior surface/%		0.39 ± 0.06

Data are presented as mean ± SD, or No. (%).

CDVA: corrected distance visual acuity, D: diopters, IOP: intraocular pressure. CCT: central corneal thickness, RST: residual stromal thickness.

In this study, the proposed model was assessed using a test set to highlight the advantages of the network architecture, with comparative experiments conducted for validation. Widely referenced models in the field, including CycleGAN, DCGAN, Pix2PixHD were employed in parallel to evaluate their performance. The efficacy of these models was rigorously compared. Additionally, an ablation experiment was performed on key structural components of the model, specifically assessing the performance of Pix2Pix, Res-Pix2Pix and Trans-Pix2Pix, to evaluate the impact of different architectural modules on the quality of the generated images. , with the average value from five random experiments taken as the experimental result. The results of these evaluations are summarized in Table 3 below.

**TABLE 3:** The performance of image generation by different generative adversarial network (GAN) models.

Model	SSIM	PSNR
CycleGAN	0.51	18.30
DCGAN	0.58	20.34
Pix2PixHD	0.63	23.46
Pix2Pix	0.64	22.97
Res-Pix2Pix	0.66	23.08
Trans-Pix2Pix	0.66	25.76
TransRes-Pix2Pix (The method in this paper)	0.67	26.02

SSIM:structural similarity index, PSNR: peak signal-to-noise ratio.

As presented in the table, the CycleGAN and DCGAN models primarily utilize unsupervised learning through adversarial training, which lacks explicit structural information and conditional constraints. This limitation makes it prone to mode collapse during training (Yoo et al., 2020; Chaurasia et al., 2024). Additionally, given the subtle and often difficult-to-discern nature of corneal features, the model struggles to capture these details effectively. Consequently, the two establish only a relatively simplistic mapping relationship, producing generated images that tend to lack diversity and exhibit significant discrepancies in detail and perceptual quality compared to the real images. Consequently, the model’s overall performance is suboptimal, suggesting that the unpaired nature of CycleGAN and DCGAN may not be well-suited for tasks requiring precise feature mapping.

In contrast, Pix2Pix benefits from paired data, where the input and output exhibit a direct, strong correlation. The content of the input image directly influences the output, allowing the generation of images that align more closely with the real scenario (Abdelmotaal et al., 2021). Unlike CycleGAN’s reliance on unpaired data, Pix2Pix shows considerable improvement in performance. Pix2PixHD, as one of the extension models of Pix2Pix, is designed to improve the task of image conversion with high resolution, and it is inclined towards multi-scale image generation (Baek et al., 2024). Therefore, in this task, it failed to fully demonstrate its advantages.

**FIGURE 5:**
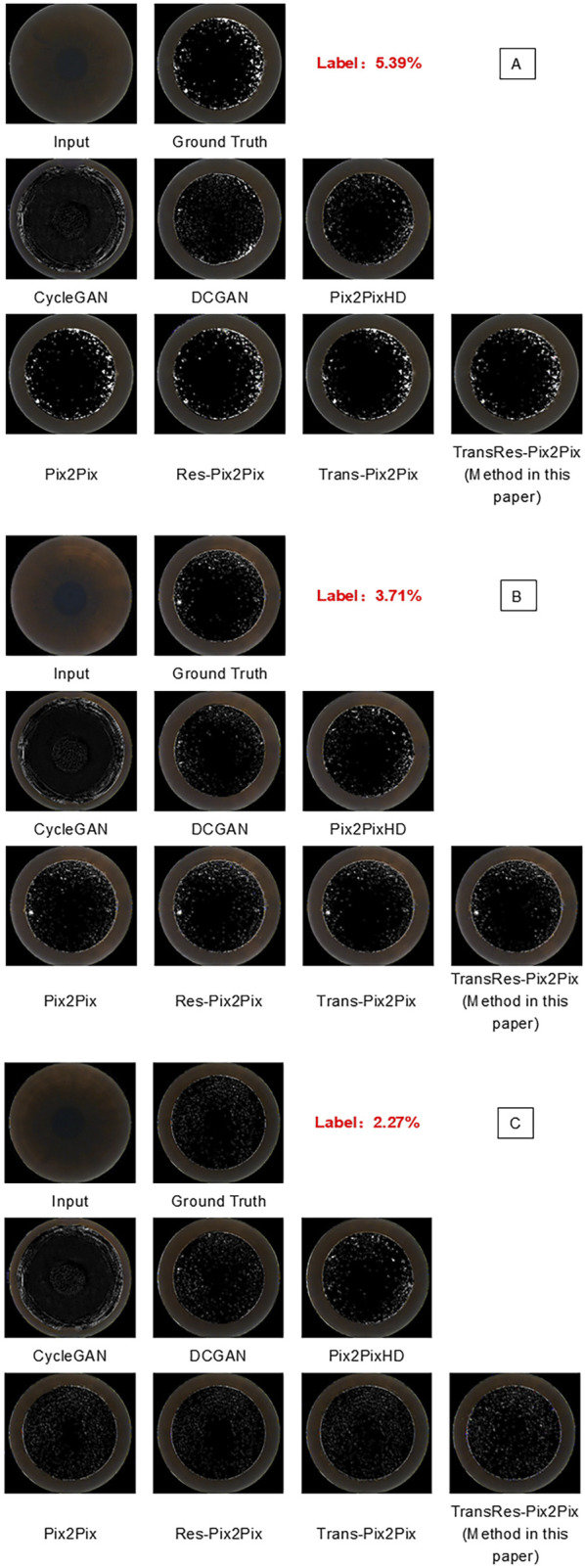
Prediction visualization results generated by various model. **(A–C)** respectively show the predicted OBL morphology images by various models from different original panoramic views of the corneal, and they are compared with the superbright OBL images processed by PS).

In particular, replacing the original structure with a residual structure in Pix2Pix enhances the model’s ability to learn complex nonlinear mappings, refining image details more accurately. This modification addresses issues like vanishing gradients, stabilizes the training process, and enables the model to capture more intricate features (Zhao B. et al., 2023; Ghanbari and Sadremomtaz, 2024). Moreover, incorporating the Transformer module within Pix2Pix introduces a self-attention mechanism that enhances the model’s understanding of the global structure of the input image. This improvement helps capture long-range dependencies and global context, leading to better image quality and improved retention of global features (Zhao B. et al., 2023; Yan et al., 2022). When both the residual structure and Transformer module are integrated into the Pix2Pix framework, the model leverages their complementary strengths in capturing both detailed and global features. This synergy significantly optimizes the model’s overall performance in image generation tasks (Zhao B. et al., 2023). Some of the generated image results are shown in Figure 5.

## Discussion

In this study, seven GAN models were systematically trained and evaluated using SSIM and PSNR as metrics to access the fidelity of generated OBL images. To determine the optimal model, an internal validation set was created, and comprehensive ablation studies were performed. Upon thorough evaluation, the model integrated within the Pix2Pix framework—enhanced by the addition of a transformer module and a residual structure—demonstrated the strongest capability for capturing critical features. It achieved an SSIM of 0.67 and a PSNR of 26.02, producing results with the highest similarity to actual intraoperative OBL images. To our knowledge, this is the first GAN model developed to predict images of intraoperative complications in corneal refractive surgery, offering potential future support for decision-making in SMILE surgeries.

OBL is a prevalent complication in SMILE and other femtosecond laser surgeries (Asif et al., 2020; He et al., 2022). Previous research has identified several influencing factors for OBL in SMILE, including central corneal thickness (CCT), corneal tissue density, corneal curvature, corneal cap thickness, lenticule thickness, residual stromal thickness (RST), laser energy, and patient astigmatism (Yang S. et al., 2023; Son et al., 2017; Brar et al., 2021; Liu et al., 2017). However, significant challenges remain in this area of study. Analyses often suffer from small sample sizes, limited inclusion criteria, and inconsistent methodologies for quantifying the OBL area. Additionally, conventional methods like linear regression fail to capture complex nonlinear relationships, limiting the development of a robust predictive model for OBL in SMILE based on preoperative parameters (El Hechi et al., 2021; Kapoor et al., 2019).

The lenticule production phase in laser scanning is a critical aspect of SMILE surgery, fully controlled by the femtosecond laser system (Carl Zeiss, VisuMax, Germany) (Titiyal et al., 2017). If complications arise during this phase, surgeons may need to immediately halt the surgery and apply alternative techniques to address the issue. This not only challenges the surgeon’s technical skills but may also increase patient anxiety, potentially affecting postoperative visual outcomes and recovery (Wan et al., 2021; Yang S. et al., 2023). Given these risks, it is crucial to accurately predict and identify potential complications before initiating the laser scan. This is particularly vital during the “negative pressure suction” step, which allows the surgeons to pause and resume the procedure without affecting the laser scan, effectively reducing surgical risks.

The occurrence of OBL typically does not result in severe outcomes; however, an extensive area of OBL can lead to significant intraoperative complications, such as difficulties in lenticule separation, epithelial breakthrough, lenticule residue, and other serious issues (Zhu et al., 2024; Sahay et al., 2021), which compromise the safety and efficacy of SMILE surgery and delay postoperative visual recovery (Aristeidou et al., 2015). Notably, these complications are more likely to arise when scanning the posterior interface of the lenticule (Son et al., 2017). A key contribution of this study is the direct capture of panoramic corneal images under negative pressure aspiration from SMILE surgery videos, providing real-time insights into corneal status and serving as input for GAN models. This method is particularly important as preoperative parameters often fail to accurately reflect the intraoperative conditions, with variables such as CCT, IOP, and others fluctuating throughout the day. This research represents the first use of a GAN model to synthesize intraoperative OBL images from preoperative corneal panoramas. Quantitative evaluation reveals that the synthesized OBL image closely matches the actual intraoperative image, with a PSNR of 26, demonstrating excellent performance, and forming a foundation for future autonomous systems and data-driven improvements in surgical techniques.

Several limitations exist in this study. Notably, no prior studies in the field of refraction have compared synthetic images of intraoperative complications with actual images to access their similarity. Consequently, quantitative metrics from other AI research in ophthalmology have been used as benchmarks. Moving forward, establishing a uniform standard for this type of evaluation is essential. Furthermore, collaboration with additional ophthalmologists for a more thorough analysis will be necessary to improve the validity and applicability of the findings. Additionally, this study focused exclusively on predicting OBL during SMILE surgery, excluding other intraoperative complications such as dark spots and aspiration. Future efforts should aim to develop a comprehensive prediction platform that incorporates all potential intraoperative complications and factors influencing postoperative visual recovery. Moreover, the research relied on an internal test set; future studies should include a broader dataset from multiple eye centers to enhance generalizability. Additionally, the practical impact of this model on SMILE surgical procedures remains unclear; further research is needed concerning how the model integrates into the Visumax system and whether the surgeon can reduce the intraoperative OBL area with the aid of the model. There is considerable potential for further exploration and refinement in this area.

## Conclusion

In conclusion, this study leverages a Pix2Pix generative adversarial network enhanced with an embedded residual module and a Transformer module, comparing various attention mechanisms to effectively genrate the morphology of OBL during SMILE surgery. This approach provides valuable insights for ophthalmologists in refining and customizing refractive surgery plans, underscoring its clinical significance and potential to improve surgical outcomes.

## Data Availability

The raw data supporting the conclusions of this article will be made available by the authors, without undue reservation.

## Abbreviations

OBL, Opaque bubble layer; SMILE, Small incision lenticule extraction; AI, Artificial intelligence; GAN, Generative adversarial networks; SSIM, Structural similarity index; PSNR, Peak signal-to-noise ratio; PS, Adobe Photoshop; D, Dioptre.
